# *N*-(3-Chloro­phen­yl)-4-nitro­benzene­sulfonamide

**DOI:** 10.1107/S1600536812047496

**Published:** 2012-11-24

**Authors:** U. Chaithanya, Sabine Foro, B. Thimme Gowda

**Affiliations:** a Department of Chemistry, Mangalore University, Mangalagangotri 574 199, Mangalore, India; b Institute of Materials Science, Darmstadt University of Technology, Petersenstrasse 23, D-64287 Darmstadt, Germany

## Abstract

There are two independent mol­ecules in the asymmetric unit of the title compound, C_12_H_9_ClN_2_O_4_S, in which the dihedral angles between the planes of the benzene rings are 46.90 (14) and 44.50 (14)°. In the crystal, N—H⋯O hydrogen bonds link the mol­ecules into zigzag chains parallel to the *a* axis.

## Related literature

For studies on the effects of substituents on the structures and other aspects of *N*-aryl­sulfonamides, see: Chaithanya *et al.* (2012 ▶); Gowda *et al.* (2002 ▶) and of *N*-chloro­aryl­amides, see: Gowda & Shetty (2004 ▶); Gowda & Weiss (1994 ▶); Shetty & Gowda (2004 ▶).
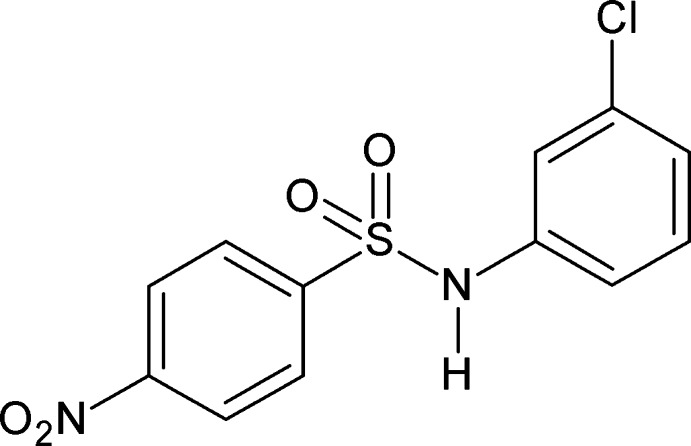


## Experimental

### 

#### Crystal data

C_12_H_9_ClN_2_O_4_S
*M* *_r_* = 312.72
Monoclinic, 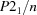

*a* = 14.3419 (8) Å
*b* = 7.7579 (4) Å
*c* = 23.895 (1) Å
β = 90.345 (5)°
*V* = 2658.6 (2) Å^3^
*Z* = 8
Mo *K*α radiation
μ = 0.46 mm^−1^
*T* = 293 K
0.48 × 0.40 × 0.20 mm

#### Data collection

Oxford Diffraction Xcalibur diffractometer with a Sapphire CCD detector
Absorption correction: multi-scan (*CrysAlis RED*; Oxford Diffraction, 2009 ▶) *T*_min_ = 0.810, *T*_max_ = 0.914
9649 measured reflections
4839 independent reflections
3112 reflections with *I* > 2σ(*I*)
*R*_int_ = 0.027

#### Refinement

*R*[*F* ^2^ > 2σ(*F* ^2^)] = 0.062
*wR*(*F* ^2^) = 0.163
*S* = 1.05
4839 reflections
367 parameters
2 restraints
H atoms treated by a mixture of independent and constrained refinement
Δρ_max_ = 0.83 e Å^−3^
Δρ_min_ = −0.29 e Å^−3^

### 

Data collection: *CrysAlis CCD* (Oxford Diffraction, 2009 ▶); cell refinement: *CrysAlis CCD*; data reduction: *CrysAlis RED* (Oxford Diffraction, 2009 ▶); program(s) used to solve structure: *SHELXS97* (Sheldrick, 2008 ▶); program(s) used to refine structure: *SHELXL97* (Sheldrick, 2008 ▶); molecular graphics: *PLATON* (Spek, 2009 ▶); software used to prepare material for publication: *SHELXL97*.

## Supplementary Material

Crystal structure: contains datablock(s) I, global. DOI: 10.1107/S1600536812047496/bt6857sup1.cif

Structure factors: contains datablock(s) I. DOI: 10.1107/S1600536812047496/bt6857Isup2.hkl

Supplementary material file. DOI: 10.1107/S1600536812047496/bt6857Isup3.cml

Additional supplementary materials:  crystallographic information; 3D view; checkCIF report

## Figures and Tables

**Table 1:** Hydrogen-bond geometry (Å, °)

*D*—H⋯*A*	*D*—H	H⋯*A*	*D*⋯*A*	*D*—H⋯*A*
N1—H1*N*⋯O8	0.87 (2)	2.22 (2)	3.052 (4)	163 (3)
N3—H3*N*⋯O4^i^	0.85 (2)	2.36 (2)	3.135 (4)	153 (4)

Symmetry code: (i) 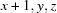.

**Fig. 1.**
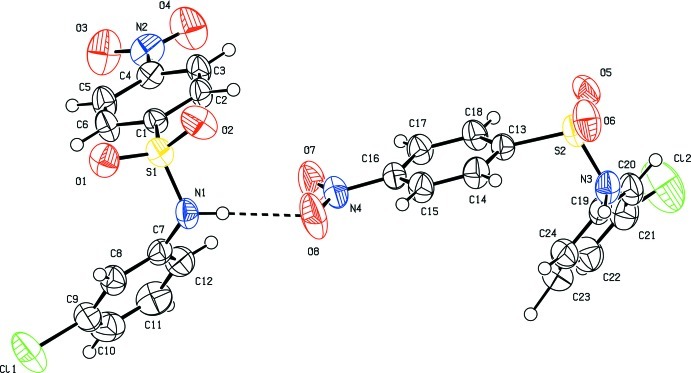
Molecular structure of the title compound, showing the atom labelling scheme and with displacement ellipsoids drawn at the 50% probability level.

**Fig. 2.**
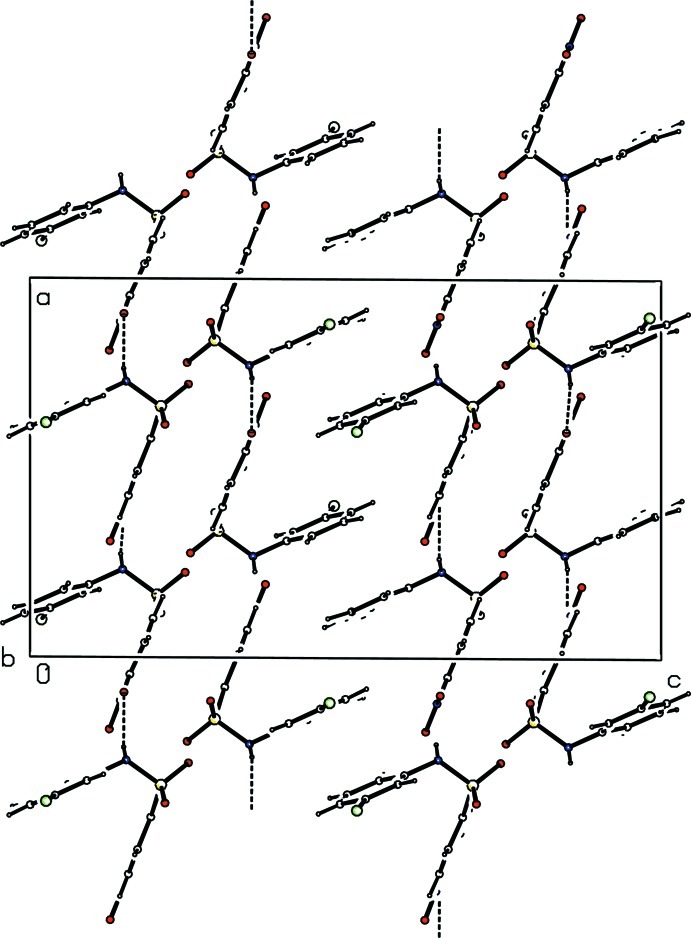
Molecular packing of the title compound with hydrogen bonding shown as dashed lines.

## supplementary crystallographic information

### Comment

As a part of studying the effect of substituents on the structures and other aspects of *N*-arylsulfonamides (Chaithanya *et al.*, 2012; Gowda *et al.*, 2002) and *N*-chloroarylamides (Gowda & Shetty, 2004; Gowda & Weiss, 1994; Shetty & Gowda, 2004), in the present work, the crystal structure of *N*-(3-chlorophenyl)-4-nitrobenzenesulfonamide (I) has been determined (Fig. 1). The asymmetric unit of the structure contains two independent molecules. The N—C bonds in the C—SO_2_—NH—C segments have *gauche* torsions with respect to the S═O bonds.

The molecules in (I) are twisted at the S—N bonds with the torsional angles of -58.67 (30) and 61.49 (30)°, compared to the value of 48.46 (18)° in *N*-(3-chlorophenyl)-2-nitrobenzenesulfonamide (II) (Chaithanya *et al.*, 2012).

The dihedral angle between the sulfonyl and the anilino rings are 46.90 (14) and 44.50 (14)°, compared to the value of 73.65 (7)° in (II).

N—H···O hydrogen bonds link the molecules into zigzag chains parallel to the *a*-axis. (Table 1, Fig. 2.)

### Experimental

The title compound was prepared by treating 4-nitrobenzenesulfonyl- chloride with 3-chloroaniline in the stoichiometric ratio and boiling the reaction mixture for 15 minutes. The reaction mixture was then cooled to room temperature and added to ice cold water (100 ml).

The resultant solid *N*-(3-chlorophenyl)-4-nitrobenzenesulfonamide was filtered under suction and washed thoroughly with cold water and dilute HCl to remove the excess sulfonylchloride and aniline, respectively. It was then recrystallized to constant melting point from dilute ethanol. The purity of the compound was checked and characterized by its infrared spectra.

Prism like colourless single crystals of the title compound used in X-ray diffraction studies were grown in ethanolic solution by slow evaporation of the solvent at room temperature.

### Refinement

H atoms bonded to C were positioned with idealized geometry using a riding model with aromatic C—H = 0.93 Å. The amino H atoms were freely refined with the N—H distance restrained to 0.86 (2) Å. All H atoms were refined with isotropic displacement parameters set at 1.2 *U*_eq_ of the parent atom. The (-1 0 3) reflection had a poor disagreement with its calculated value and was omitted from the refinement.

### Crystal data

**Table:**

C_12_H_9_ClN_2_O_4_S	*F*(000) = 1280
*M**_r_* = 312.72	*D*_x_ = 1.563 Mg m^−^^3^
Monoclinic, *P*2_1_/*n*	Mo *K*α radiation, λ = 0.71073 Å
Hall symbol: -P 2yn	Cell parameters from 3273 reflections
*a* = 14.3419 (8) Å	θ = 2.6–27.8°
*b* = 7.7579 (4) Å	µ = 0.46 mm^−^^1^
*c* = 23.895 (1) Å	*T* = 293 K
β = 90.345 (5)°	Prism, colourless
*V* = 2658.6 (2) Å^3^	0.48 × 0.40 × 0.20 mm
*Z* = 8	

### Data collection

**Table:**

Oxford Diffraction Xcalibur diffractometer with a Sapphire CCD detector	4839 independent reflections
Radiation source: fine-focus sealed tube	3112 reflections with *I* > 2σ(*I*)
Graphite monochromator	*R*_int_ = 0.027
Rotation method data acquisition using ω scans	θ_max_ = 25.4°, θ_min_ = 2.8°
Absorption correction: multi-scan (*CrysAlis RED*; Oxford Diffraction, 2009)	*h* = −9→17
*T*_min_ = 0.810, *T*_max_ = 0.914	*k* = −9→5
9649 measured reflections	*l* = −27→28

### Refinement

**Table:**

Refinement on *F*^2^	Primary atom site location: structure-invariant direct methods
Least-squares matrix: full	Secondary atom site location: difference Fourier map
*R*[*F*^2^ > 2σ(*F*^2^)] = 0.062	Hydrogen site location: inferred from neighbouring sites
*wR*(*F*^2^) = 0.163	H atoms treated by a mixture of independent and constrained refinement
*S* = 1.05	*w* = 1/[σ^2^(*F*_o_^2^) + (0.0682*P*)^2^ + 2.2606*P*] where *P* = (*F*_o_^2^ + 2*F*_c_^2^)/3
4839 reflections	(Δ/σ)_max_ = 0.009
367 parameters	Δρ_max_ = 0.83 e Å^−^^3^
2 restraints	Δρ_min_ = −0.29 e Å^−^^3^

### Special details

**Table:**

Experimental. CrysAlis RED (Oxford Diffraction, 2009) Empirical absorption correction using spherical harmonics, implemented in SCALE3 ABSPACK scaling algorithm.
Geometry. All e.s.d.'s (except the e.s.d. in the dihedral angle between two l.s. planes) are estimated using the full covariance matrix. The cell e.s.d.'s are taken into account individually in the estimation of e.s.d.'s in distances, angles and torsion angles; correlations between e.s.d.'s in cell parameters are only used when they are defined by crystal symmetry. An approximate (isotropic) treatment of cell e.s.d.'s is used for estimating e.s.d.'s involving l.s. planes.
Refinement. Refinement of *F*^2^ against ALL reflections. The weighted *R*-factor *wR* and goodness of fit *S* are based on *F*^2^, conventional *R*-factors *R* are based on *F*, with *F* set to zero for negative *F*^2^. The threshold expression of *F*^2^ > σ(*F*^2^) is used only for calculating *R*-factors(gt) *etc*. and is not relevant to the choice of reflections for refinement. *R*-factors based on *F*^2^ are statistically about twice as large as those based on *F*, and *R*- factors based on ALL data will be even larger.

### Fractional atomic coordinates and isotropic or equivalent isotropic displacement parameters (Å^2^)

**Table:**

	*x*	*y*	*z*	*U*_iso_*/*U*_eq_	
Cl1	0.09787 (10)	−0.35505 (17)	0.01810 (6)	0.0946 (5)	
S1	0.16559 (6)	0.12543 (12)	0.20178 (4)	0.0434 (3)	
O1	0.11688 (19)	−0.0333 (3)	0.20733 (11)	0.0562 (7)	
O2	0.22431 (18)	0.1878 (4)	0.24582 (11)	0.0627 (8)	
O3	−0.1904 (2)	0.6207 (4)	0.12567 (15)	0.0788 (10)	
O4	−0.0928 (2)	0.8146 (4)	0.14880 (15)	0.0823 (10)	
N1	0.23212 (19)	0.1114 (4)	0.14670 (14)	0.0452 (8)	
H1N	0.272 (2)	0.194 (4)	0.1439 (15)	0.054*	
N2	−0.1143 (2)	0.6643 (5)	0.14311 (14)	0.0566 (9)	
C1	0.0817 (2)	0.2853 (4)	0.18560 (14)	0.0353 (8)	
C2	0.1026 (2)	0.4570 (4)	0.19499 (15)	0.0423 (9)	
H2	0.1599	0.4877	0.2104	0.051*	
C3	0.0387 (2)	0.5819 (4)	0.18147 (15)	0.0442 (9)	
H3	0.0514	0.6978	0.1878	0.053*	
C4	−0.0450 (2)	0.5309 (4)	0.15819 (14)	0.0405 (8)	
C5	−0.0679 (2)	0.3612 (5)	0.14868 (16)	0.0500 (10)	
H5	−0.1253	0.3313	0.1332	0.060*	
C6	−0.0030 (2)	0.2365 (5)	0.16275 (16)	0.0469 (9)	
H6	−0.0163	0.1205	0.1569	0.056*	
C7	0.1911 (2)	0.0681 (5)	0.09334 (15)	0.0413 (9)	
C8	0.1675 (2)	−0.1024 (5)	0.08276 (17)	0.0482 (10)	
H8	0.1777	−0.1874	0.1095	0.058*	
C9	0.1285 (3)	−0.1422 (5)	0.03155 (18)	0.0544 (10)	
C10	0.1144 (3)	−0.0199 (7)	−0.00864 (18)	0.0658 (12)	
H10	0.0883	−0.0494	−0.0431	0.079*	
C11	0.1395 (3)	0.1489 (6)	0.00264 (18)	0.0668 (12)	
H11	0.1305	0.2334	−0.0245	0.080*	
C12	0.1773 (3)	0.1921 (5)	0.05326 (17)	0.0549 (10)	
H12	0.1937	0.3058	0.0606	0.066*	
Cl2	0.61875 (14)	1.53899 (18)	0.02541 (7)	0.1192 (6)	
S2	0.66256 (6)	1.03064 (12)	0.20747 (4)	0.0478 (3)	
O5	0.61482 (19)	1.1899 (3)	0.21387 (12)	0.0583 (7)	
O6	0.72154 (19)	0.9650 (4)	0.25102 (12)	0.0703 (9)	
O7	0.3061 (2)	0.5453 (4)	0.12695 (15)	0.0789 (10)	
O8	0.4020 (2)	0.3483 (4)	0.15000 (17)	0.0907 (11)	
N3	0.7282 (2)	1.0461 (4)	0.15232 (15)	0.0503 (8)	
H3N	0.762 (2)	0.958 (4)	0.1481 (16)	0.060*	
N4	0.3811 (2)	0.4986 (5)	0.14497 (14)	0.0568 (9)	
C13	0.5779 (2)	0.8725 (4)	0.19141 (14)	0.0373 (8)	
C14	0.5992 (2)	0.6997 (5)	0.19794 (16)	0.0470 (9)	
H14	0.6570	0.6670	0.2122	0.056*	
C15	0.5345 (2)	0.5764 (5)	0.18336 (16)	0.0486 (10)	
H15	0.5474	0.4598	0.1880	0.058*	
C16	0.4501 (2)	0.6303 (5)	0.16172 (15)	0.0421 (9)	
C17	0.4269 (2)	0.8010 (5)	0.15583 (15)	0.0464 (9)	
H17	0.3685	0.8331	0.1423	0.056*	
C18	0.4919 (2)	0.9235 (5)	0.17029 (15)	0.0437 (9)	
H18	0.4783	1.0400	0.1660	0.052*	
C19	0.6866 (2)	1.0996 (5)	0.10019 (16)	0.0448 (9)	
C20	0.6731 (3)	1.2725 (5)	0.08980 (18)	0.0516 (10)	
H20	0.6901	1.3547	0.1163	0.062*	
C21	0.6343 (3)	1.3211 (5)	0.0398 (2)	0.0633 (12)	
C22	0.6084 (3)	1.2024 (6)	0.00016 (19)	0.0672 (12)	
H22	0.5809	1.2385	−0.0332	0.081*	
C23	0.6231 (3)	1.0320 (6)	0.0097 (2)	0.0692 (13)	
H23	0.6067	0.9511	−0.0173	0.083*	
C24	0.6630 (3)	0.9787 (5)	0.06058 (19)	0.0592 (11)	
H24	0.6734	0.8623	0.0674	0.071*	

### Atomic displacement parameters (Å^2^)

**Table:**

	*U*^11^	*U*^22^	*U*^33^	*U*^12^	*U*^13^	*U*^23^
Cl1	0.1078 (11)	0.0610 (8)	0.1148 (11)	−0.0144 (7)	−0.0227 (9)	−0.0310 (8)
S1	0.0416 (5)	0.0361 (5)	0.0524 (6)	0.0073 (4)	−0.0107 (4)	0.0001 (4)
O1	0.0621 (17)	0.0330 (15)	0.0736 (19)	0.0044 (13)	0.0006 (14)	0.0137 (13)
O2	0.0595 (17)	0.0634 (19)	0.0648 (18)	0.0187 (15)	−0.0274 (14)	−0.0094 (15)
O3	0.0481 (18)	0.075 (2)	0.113 (3)	0.0106 (16)	−0.0208 (18)	0.0294 (19)
O4	0.087 (2)	0.0378 (18)	0.122 (3)	0.0197 (17)	−0.020 (2)	0.0079 (18)
N1	0.0314 (16)	0.0350 (18)	0.069 (2)	−0.0020 (13)	−0.0051 (15)	−0.0042 (16)
N2	0.056 (2)	0.053 (2)	0.061 (2)	0.0157 (18)	0.0009 (17)	0.0160 (18)
C1	0.0341 (18)	0.0297 (19)	0.042 (2)	0.0035 (15)	−0.0032 (15)	−0.0025 (16)
C2	0.0317 (18)	0.038 (2)	0.057 (2)	−0.0035 (16)	−0.0079 (16)	−0.0043 (18)
C3	0.046 (2)	0.0253 (19)	0.061 (2)	−0.0012 (16)	−0.0008 (19)	0.0000 (17)
C4	0.0397 (19)	0.035 (2)	0.047 (2)	0.0074 (16)	0.0010 (16)	0.0079 (17)
C5	0.038 (2)	0.045 (2)	0.067 (3)	−0.0046 (17)	−0.0137 (18)	−0.002 (2)
C6	0.042 (2)	0.033 (2)	0.066 (3)	−0.0026 (17)	−0.0122 (19)	−0.0047 (18)
C7	0.0292 (18)	0.040 (2)	0.055 (2)	0.0028 (16)	0.0045 (16)	−0.0034 (18)
C8	0.043 (2)	0.037 (2)	0.065 (3)	0.0010 (17)	0.0002 (19)	0.0002 (19)
C9	0.046 (2)	0.050 (3)	0.066 (3)	−0.0033 (19)	0.001 (2)	−0.013 (2)
C10	0.061 (3)	0.083 (4)	0.053 (3)	0.007 (3)	−0.002 (2)	−0.009 (3)
C11	0.074 (3)	0.069 (3)	0.057 (3)	0.007 (3)	0.001 (2)	0.013 (2)
C12	0.059 (3)	0.042 (2)	0.063 (3)	0.001 (2)	0.007 (2)	0.002 (2)
Cl2	0.1649 (16)	0.0543 (8)	0.1379 (14)	−0.0001 (9)	−0.0415 (12)	0.0214 (9)
S2	0.0420 (5)	0.0396 (6)	0.0615 (6)	−0.0079 (4)	−0.0109 (5)	−0.0029 (5)
O5	0.0621 (17)	0.0346 (15)	0.0783 (19)	−0.0044 (13)	0.0024 (14)	−0.0155 (14)
O6	0.0623 (18)	0.072 (2)	0.0765 (19)	−0.0191 (16)	−0.0324 (16)	0.0079 (16)
O7	0.0486 (17)	0.076 (2)	0.112 (3)	−0.0156 (16)	−0.0221 (18)	−0.0165 (19)
O8	0.083 (2)	0.0390 (19)	0.150 (3)	−0.0190 (17)	−0.019 (2)	−0.006 (2)
N3	0.0324 (17)	0.0358 (19)	0.083 (2)	0.0028 (13)	−0.0023 (16)	0.0026 (17)
N4	0.052 (2)	0.050 (2)	0.068 (2)	−0.0148 (18)	−0.0021 (18)	−0.0091 (19)
C13	0.0332 (18)	0.0322 (19)	0.046 (2)	0.0006 (15)	−0.0018 (15)	−0.0003 (16)
C14	0.037 (2)	0.041 (2)	0.063 (2)	0.0035 (17)	−0.0058 (18)	0.0061 (19)
C15	0.045 (2)	0.030 (2)	0.071 (3)	0.0015 (17)	0.002 (2)	0.0024 (18)
C16	0.0378 (19)	0.039 (2)	0.049 (2)	−0.0060 (17)	0.0010 (17)	−0.0050 (17)
C17	0.0353 (19)	0.045 (2)	0.058 (2)	0.0016 (17)	−0.0088 (17)	−0.0015 (19)
C18	0.043 (2)	0.0287 (19)	0.060 (2)	0.0035 (16)	−0.0083 (18)	0.0019 (17)
C19	0.0335 (19)	0.039 (2)	0.062 (3)	−0.0053 (16)	0.0090 (18)	0.0008 (19)
C20	0.046 (2)	0.034 (2)	0.075 (3)	−0.0039 (17)	−0.001 (2)	−0.001 (2)
C21	0.066 (3)	0.043 (2)	0.082 (3)	−0.003 (2)	0.000 (2)	0.013 (2)
C22	0.071 (3)	0.064 (3)	0.066 (3)	−0.005 (3)	−0.006 (2)	0.001 (3)
C23	0.071 (3)	0.064 (3)	0.073 (3)	−0.011 (3)	0.011 (3)	−0.023 (3)
C24	0.057 (3)	0.041 (2)	0.080 (3)	−0.005 (2)	0.012 (2)	−0.012 (2)

### Geometric parameters (Å, º)

**Table:**

Cl1—C9	1.738 (4)	Cl2—C21	1.739 (4)
S1—O1	1.423 (3)	S2—O5	1.421 (3)
S1—O2	1.428 (3)	S2—O6	1.431 (3)
S1—N1	1.634 (3)	S2—N3	1.629 (3)
S1—C1	1.770 (3)	S2—C13	1.767 (3)
O3—N2	1.215 (4)	O7—N4	1.211 (4)
O4—N2	1.214 (4)	O8—N4	1.209 (4)
N1—C7	1.441 (5)	N3—C19	1.439 (5)
N1—H1N	0.865 (18)	N3—H3N	0.845 (18)
N2—C4	1.478 (4)	N4—C16	1.476 (4)
C1—C6	1.382 (4)	C13—C14	1.383 (5)
C1—C2	1.383 (5)	C13—C18	1.388 (5)
C2—C3	1.371 (5)	C14—C15	1.376 (5)
C2—H2	0.9300	C14—H14	0.9300
C3—C4	1.377 (5)	C15—C16	1.379 (5)
C3—H3	0.9300	C15—H15	0.9300
C4—C5	1.375 (5)	C16—C17	1.373 (5)
C5—C6	1.382 (5)	C17—C18	1.373 (5)
C5—H5	0.9300	C17—H17	0.9300
C6—H6	0.9300	C18—H18	0.9300
C7—C12	1.371 (5)	C19—C24	1.373 (5)
C7—C8	1.388 (5)	C19—C20	1.378 (5)
C8—C9	1.377 (5)	C20—C21	1.368 (6)
C8—H8	0.9300	C20—H20	0.9300
C9—C10	1.364 (6)	C21—C22	1.371 (6)
C10—C11	1.384 (6)	C22—C23	1.358 (6)
C10—H10	0.9300	C22—H22	0.9300
C11—C12	1.364 (6)	C23—C24	1.402 (6)
C11—H11	0.9300	C23—H23	0.9300
C12—H12	0.9300	C24—H24	0.9300
			
O1—S1—O2	120.83 (17)	O5—S2—O6	120.94 (18)
O1—S1—N1	107.88 (16)	O5—S2—N3	107.70 (17)
O2—S1—N1	105.75 (17)	O6—S2—N3	105.83 (18)
O1—S1—C1	107.07 (16)	O5—S2—C13	107.24 (16)
O2—S1—C1	108.70 (16)	O6—S2—C13	108.26 (17)
N1—S1—C1	105.68 (16)	N3—S2—C13	105.98 (16)
C7—N1—S1	119.4 (2)	C19—N3—S2	118.9 (2)
C7—N1—H1N	112 (3)	C19—N3—H3N	111 (3)
S1—N1—H1N	114 (3)	S2—N3—H3N	112 (3)
O3—N2—O4	122.2 (3)	O8—N4—O7	122.9 (3)
O3—N2—C4	119.4 (4)	O8—N4—C16	118.3 (3)
O4—N2—C4	118.4 (3)	O7—N4—C16	118.8 (4)
C6—C1—C2	121.2 (3)	C14—C13—C18	120.8 (3)
C6—C1—S1	119.3 (3)	C14—C13—S2	119.9 (3)
C2—C1—S1	119.5 (3)	C18—C13—S2	119.3 (3)
C3—C2—C1	119.9 (3)	C15—C14—C13	119.9 (3)
C3—C2—H2	120.1	C15—C14—H14	120.1
C1—C2—H2	120.1	C13—C14—H14	120.1
C2—C3—C4	118.1 (3)	C14—C15—C16	118.2 (3)
C2—C3—H3	120.9	C14—C15—H15	120.9
C4—C3—H3	120.9	C16—C15—H15	120.9
C5—C4—C3	123.2 (3)	C17—C16—C15	122.8 (3)
C5—C4—N2	118.0 (3)	C17—C16—N4	118.6 (3)
C3—C4—N2	118.7 (3)	C15—C16—N4	118.5 (3)
C4—C5—C6	118.0 (3)	C16—C17—C18	118.6 (3)
C4—C5—H5	121.0	C16—C17—H17	120.7
C6—C5—H5	121.0	C18—C17—H17	120.7
C1—C6—C5	119.5 (3)	C17—C18—C13	119.6 (3)
C1—C6—H6	120.2	C17—C18—H18	120.2
C5—C6—H6	120.2	C13—C18—H18	120.2
C12—C7—C8	120.5 (4)	C24—C19—C20	120.5 (4)
C12—C7—N1	120.7 (3)	C24—C19—N3	120.0 (4)
C8—C7—N1	118.8 (3)	C20—C19—N3	119.6 (4)
C9—C8—C7	118.2 (4)	C21—C20—C19	118.8 (4)
C9—C8—H8	120.9	C21—C20—H20	120.6
C7—C8—H8	120.9	C19—C20—H20	120.6
C10—C9—C8	121.8 (4)	C20—C21—C22	121.7 (4)
C10—C9—Cl1	119.6 (3)	C20—C21—Cl2	119.4 (4)
C8—C9—Cl1	118.6 (3)	C22—C21—Cl2	118.8 (4)
C9—C10—C11	118.9 (4)	C23—C22—C21	119.8 (4)
C9—C10—H10	120.5	C23—C22—H22	120.1
C11—C10—H10	120.5	C21—C22—H22	120.1
C12—C11—C10	120.4 (4)	C22—C23—C24	119.7 (4)
C12—C11—H11	119.8	C22—C23—H23	120.2
C10—C11—H11	119.8	C24—C23—H23	120.2
C11—C12—C7	120.1 (4)	C19—C24—C23	119.6 (4)
C11—C12—H12	119.9	C19—C24—H24	120.2
C7—C12—H12	119.9	C23—C24—H24	120.2
			
O1—S1—N1—C7	55.6 (3)	O5—S2—N3—C19	−53.0 (3)
O2—S1—N1—C7	−173.8 (3)	O6—S2—N3—C19	176.3 (3)
C1—S1—N1—C7	−58.7 (3)	C13—S2—N3—C19	61.5 (3)
O1—S1—C1—C6	−21.6 (3)	O5—S2—C13—C14	−162.2 (3)
O2—S1—C1—C6	−153.6 (3)	O6—S2—C13—C14	−30.2 (4)
N1—S1—C1—C6	93.2 (3)	N3—S2—C13—C14	83.0 (3)
O1—S1—C1—C2	159.7 (3)	O5—S2—C13—C18	20.2 (3)
O2—S1—C1—C2	27.6 (3)	O6—S2—C13—C18	152.3 (3)
N1—S1—C1—C2	−85.5 (3)	N3—S2—C13—C18	−94.6 (3)
C6—C1—C2—C3	−0.1 (6)	C18—C13—C14—C15	0.1 (6)
S1—C1—C2—C3	178.6 (3)	S2—C13—C14—C15	−177.5 (3)
C1—C2—C3—C4	−0.6 (5)	C13—C14—C15—C16	0.9 (6)
C2—C3—C4—C5	1.0 (6)	C14—C15—C16—C17	−2.1 (6)
C2—C3—C4—N2	−179.6 (3)	C14—C15—C16—N4	178.8 (3)
O3—N2—C4—C5	3.8 (5)	O8—N4—C16—C17	179.6 (4)
O4—N2—C4—C5	−176.4 (4)	O7—N4—C16—C17	−0.5 (5)
O3—N2—C4—C3	−175.6 (4)	O8—N4—C16—C15	−1.3 (5)
O4—N2—C4—C3	4.2 (5)	O7—N4—C16—C15	178.6 (4)
C3—C4—C5—C6	−0.7 (6)	C15—C16—C17—C18	2.3 (6)
N2—C4—C5—C6	179.9 (3)	N4—C16—C17—C18	−178.6 (3)
C2—C1—C6—C5	0.4 (6)	C16—C17—C18—C13	−1.3 (6)
S1—C1—C6—C5	−178.3 (3)	C14—C13—C18—C17	0.2 (6)
C4—C5—C6—C1	0.0 (6)	S2—C13—C18—C17	177.7 (3)
S1—N1—C7—C12	104.1 (4)	S2—N3—C19—C24	−99.5 (4)
S1—N1—C7—C8	−77.1 (4)	S2—N3—C19—C20	82.3 (4)
C12—C7—C8—C9	−1.1 (5)	C24—C19—C20—C21	1.2 (6)
N1—C7—C8—C9	−179.9 (3)	N3—C19—C20—C21	179.3 (3)
C7—C8—C9—C10	1.1 (6)	C19—C20—C21—C22	0.3 (6)
C7—C8—C9—Cl1	−179.5 (3)	C19—C20—C21—Cl2	−178.8 (3)
C8—C9—C10—C11	−0.4 (6)	C20—C21—C22—C23	−1.5 (7)
Cl1—C9—C10—C11	−179.8 (3)	Cl2—C21—C22—C23	177.6 (4)
C9—C10—C11—C12	−0.3 (7)	C21—C22—C23—C24	1.2 (7)
C10—C11—C12—C7	0.3 (6)	C20—C19—C24—C23	−1.4 (6)
C8—C7—C12—C11	0.4 (6)	N3—C19—C24—C23	−179.5 (3)
N1—C7—C12—C11	179.2 (3)	C22—C23—C24—C19	0.2 (6)

### Hydrogen-bond geometry (Å, º)

**Table:**

*D*—H···*A*	*D*—H	H···*A*	*D*···*A*	*D*—H···*A*
N1—H1*N*···O8	0.87 (2)	2.22 (2)	3.052 (4)	163 (3)
N3—H3*N*···O4^i^	0.85 (2)	2.36 (2)	3.135 (4)	153 (4)

Symmetry code: (i) *x*+1, *y*, *z*.
